# Association between thyroid hormone and components of metabolic syndrome in euthyroid Korean adults: A population-based study

**DOI:** 10.1097/MD.0000000000028409

**Published:** 2021-12-23

**Authors:** Kyung A. Shin, Eun Jae Kim

**Affiliations:** a Clinical Laboratory Science, Shinsung University, Daehak-ro, Jeongmi-myeon, Dangjin-si, Chungcheongnam-do, Republic of Korea; b Medical Laboratory Science, Jeonju Kijeon University, Wansan-gu, Jeonju-si Jeollabuk-do, Republic of Korea.

**Keywords:** euthyroid, Korean adults, metabolic syndrome, thyroid hormone

## Abstract

Thyroid dysfunction increases the prevalence of metabolic syndrome. However, the link between thyroid hormones and metabolic syndrome remains debatable, and the effect of sex on their relationship is not completely understood. To elucidate the relationship of thyroid hormones with metabolic syndrome and its components according to sex in euthyroid individuals in South Korea. Adult participants who underwent thyroid tests at our institution between January 2015 and December 2018 and had thyroid-stimulating hormone (TSH; 0.270–4.200 μIU/mL) and free thyroxine (FT4; 0.93–1.70 ng/dL) levels in the normal range were included. After correcting for age and body mass index, multiple linear regression was performed to assess the association of TSH and FT4 with metabolic syndrome and its components, and logistic regression was performed to estimate the risk of developing metabolic syndrome and its components according to different thyroid hormone quartiles. We included 12,478 men and 7,575 women in this study. The prevalence of metabolic syndrome was 9.68%. In men, TSH was positively associated with blood pressure and triglycerides, and the odds ratio for high blood pressure and hypertriglyceridemia was approximately 1.3 times higher in the fourth quartile than in the first quartile. FT4 associated positively with waist circumference, and a high odds ratio for abdominal obesity in the fourth quartile was observed in both men (odds ratio [OR], 1.239; 95% confidence interval [CI], 1.045–1.470) and women (OR, 1.302; 95% CI, 1.029–1.649). A negative association was found between FT4 and triglycerides, and concurrently, the odds ratios for hypertriglyceridemia were lower in the fourth quartile in both men (OR, 0.692; 95% CI, 0.619–0.774) and women (OR: 0.641; 95% CI: 0.512–0.803). In addition, a higher odds ratio for high blood pressure was observed in the fourth quartiles of FT4 and TSH in women. However, there was no association between TSH and FT4 levels and the onset of metabolic syndrome in either of the sexes. Serum TSH and FT4 levels were associated with different metabolic syndrome components in men and women, but there was no association with the onset of metabolic syndrome.

## 1 Introduction

Metabolic syndrome is one of the endocrine disorders characterized by a cluster of cardiovascular risk factors such as abdominal obesity, dyslipidemia, hyperglycemia, and hypertension.^[1]^ Metabolic syndrome is closely associated with a high risk of cardiovascular diseases and type 2 diabetes.^[2,3]^ According to the data published by the International Diabetes Federation, one in four people in the world are diagnosed with metabolic syndrome.^[4]^ In Korea, its prevalence was reported to be ∼28.2% in 2012.^[5]^ Thus, metabolic syndrome is emerging as a serious public health problem worldwide.^[6]^

Thyroid disease is the most common endocrine disease, and more than 300 million people are reported to have thyroid dysfunction globally.^[7]^ Thyroid hormones are critical in regulating energy balance and metabolism.^[8]^ Thyroid dysfunction causes insulin resistance and affects glucose and lipid metabolism, thereby increasing the prevalence of metabolic syndrome.^[8–10]^ In a study on the relationship between thyroid hormone and metabolic syndrome in euthyroid subjects, Ruhla et al^[11]^ reported that a thyroid-stimulating hormone (TSH) level of 2.5 to 4.5 mU/L in was associated with an increased likelihood of having metabolic syndrome and that TSH below 2.5 mU/L was associated with a favorable metabolic profile, although this relationship should be investigated further. However, a study by Mehran et al^[12]^ reported that free thyroxine (FT4), not TSH, was related to the occurrence of metabolic syndrome and its components. Kim et al^[13]^ reported that there was no association between serum FT4 levels and metabolic syndrome after correcting for age. Although consistent results have been reported on the relationship between abnormal thyroid hormones and metabolic syndrome in these reported results, the relationship between the normal range of thyroid hormones and metabolic syndrome is still controversial. In addition, it has been reported that the prevalence of metabolic syndrome components and their association with thyroid hormones vary according to sex.^[14–16]^ Regardless, many studies have not considered sex as a factor when investigating the relationship between thyroid function and the components of metabolic syndrome.

Various thyroid diseases are associated with excessive and insufficient iodine intake. Koreans consume high amounts of seaweed and seafood, showing a characteristically high intake of iodine.^[17,18]^ Therefore, this study aimed to investigate the relationship of thyroid hormones with metabolic syndrome and its components according to sex in euthyroid South Koreans.

## 2 Materials and methods

### 2.1 Participants

**Figure 1:**
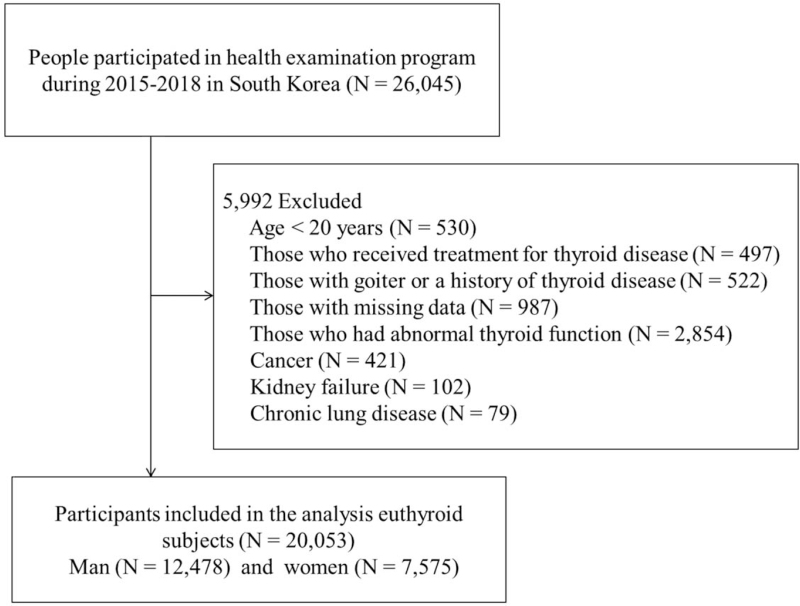
Flow chart of the study population.

This study included patients between 20 and 80 years who underwent thyroid tests at the health examination center of a general hospital in Gyeonggi Province, Korea, between January 2015 and December 2018. In total, 22,907 participants were included. We excluded those who received treatment for thyroid disease; those with goiter or a history of thyroid disease, cancer, kidney failure, or chronic lung disease; and those with missing information. The diseases that comprised the exclusion criteria were identified from the diagnosis mentioned on the patients chart. Among the total population, 20,053 participants with TSH (0.270–4.200 μIU/mL) and FT4 (0.93–1.70 ng/dL) levels in the normal range, related to euthyroidism, were selected as the final participants for the study (Fig. 1). Data on present and past medical history were collected from 12,478 men and 7,575 women through questionnaires. This study was conducted after review and approval by the Institutional Review Board of Bundang Jesaeng Hospital, Gyeonggi-do, Korea (DMC 2021-03-003). Requirement for written informed consent was waived by the institutional ethics committee.

### 2.2 Anthropometry and blood pressure measurement

The height and body weight of the participants were measured using a body composition analyzer (Inbody 720 [Biospace, Seoul, Korea]), and the body mass index was calculated using the formula: body weight (kg)/height (m)^2^. Waist circumference was measured with a tape measuring around the midpoint between the lowermost part of the ribs and the highest part of the pelvis after exhaling, with the body weight evenly distributed and participants’ feet placed 25 to 30 cm apart. Blood pressure was measured with an automatic sphygmomanometer (HBP-9020 [OMRON, Kyoto, Japan]), and measurements were taken with the patient in a sitting position after a 10-minute rest period. The anthropometric measurements and blood pressure measurements were performed on the same day as that of the blood test.

### 2.3 Diagnostic criteria and blood analysis

The diagnostic criteria for metabolic syndrome were defined according to the Adult Treatment Panel III of the National Cholesterol Education Program.^[19]^ The components of the metabolic syndrome included a fasting blood glucose level ≥110 mg/dL, blood triglyceride level ≥150 mg/dL, blood high-density lipoprotein (HDL)-cholesterol level <40 mg/dL for men and <50 mg/dL for women, systolic blood pressure ≥130 mm Hg or diastolic blood pressure ≥85 mm Hg, and waist circumference ≥90 and ≥80 cm for men and women, respectively, according to Asia-Pacific standards.^[20]^ A diagnosis of metabolic syndrome was made when three or more of these components exceeded the standard values. To perform laboratory blood tests, blood samples were collected from the median antebrachial vein after the patient fasted for at least 8 hours. For hematologic analysis, levels of total cholesterol, triglycerides, HDL-cholesterol, low-density lipoprotein (LDL)-cholesterol, fasting glucose, and uric acid were measured using TBA-2000FR NEO (Toshiba, Tokyo, Japan). Hemoglobin A1c (HbA1c) was measured using Variant II (Bio-Rad, CA), according to the principle of high-performance liquid chromatography. Insulin, TSH, and FT4 levels were measured using Roche Modular Analytics E170 (Mannheim, Germany) according to the principle of electrochemiluminescence immunoassay. The homeostasis model assessment of insulin resistance was calculated using the following formula: fasting insulin (μIU/mL) × fasting glucose (mg/dL)/405].^[21]^

### 2.4 Statistical analysis

The data obtained in this study were analyzed using IBM SPSS Statistics 24.0 (IBM, NY). Descriptive statistics are presented as the mean ± standard deviation. An independent-samples *t* test was performed to verify the differences in anthropometric and hematological variables according to the presence or absence of metabolic syndrome between men and women. One way-ANOVA was performed to verify the differences in anthropometric and hematological variables according to TSH and FT4 quartiles between sexes. A chi-square test was performed to confirm the difference in the prevalence of metabolic syndrome according to the TSH and FT4 quartiles.

The quartiles were defined as follows: Men TSH Q1; <1.190, Q2; 1.190–1.704, Q3; 1.705–2.389, Q4; 2.390 or higher/Men FT4 Q1; <1.220, Q2; 1.220–1.329, Q3; 1.330–1.439, Q4; 1.440 or higher/Women TSH Q1; <1.310, Q2; 1.310–1.889, Q3; 1.890–2.599, Q4; 2.600 or higher/Women FT4 Q1; <1.130, Q2; 1.130–1.299, Q3; 1.230–1.329, Q4; 1.330 or higher. In addition, in order to evaluate the relationship between TSH and FT4 and metabolic syndrome components, multiple linear regression analysis was performed after correcting for age and body mass index as independent variables and TSH and FT4 as dependent variables of metabolic syndrome and its components. For evaluating the risk of metabolic syndrome and its components according to the TSH and FT4 quartiles, the odds ratios (ORs) and 95% confidence intervals (CIs) were calculated using binary logistic regression after correcting for age and body mass index. Statistical significance was set at *P *<* *.05.

## 3 Results

### 3.1 Medical characteristics of subjects with diagnosis of metabolic syndrome

The baseline characteristics of the study participants are listed in Table 1. The prevalence of metabolic syndrome was 9.68% (n = 1941), and the average age of the participants was 45.1 ± 11.0 years. Participants were classified into the metabolic syndrome group and the non-metabolic syndrome group by sex. In men, anthropometric and hematologic indicators, excluding LDL-cholesterol level, were significantly different between both groups. Among the indicators, HDL-cholesterol and FT4 levels were higher in the non-metabolic syndrome group than in the other group; however, other indicators were higher in the metabolic syndrome group than in the other group (all *P* < .001 except TSH [*P* = 0.037]). In women, there was a significant difference between the both groups regarding all indicators except TSH level, and all indicators except HDL-cholesterol and FT4 levels were higher in the metabolic syndrome group than in the non-metabolic syndrome group (all *P* < .001 except FT4 [*P* = .001]).

**Table 1:** Anthropometric and biochemical characteristics of the study subjects according to sex.

	Men (n = 12,478)			Women (n = 7,575)		
Variables	MetS (N = 1,462)	Non-MetS (N = 11,016)	*P*	MetS (N = 479)	Non-MetS (N = 7,096)	*P*
Age (years)	48.7 ± 10.9	45.3 ± 10.8	<.001	55.6 ± 11.0	43.6 ± 11.0	<.001
Weight (kg)	81.6 ± 10.9	71.4 ± 9.6	<.001	65.7 ± 9.2	56.0 ± 7.6	<.001
BMI (kg/m^2^)	27.7 ± 2.9	24.3 ± 2.7	<.001	27.0 ± 3.0	22.1 ± 3.0	<.001
WC (cm)	92.3 ± 6.7	82.6 ± 7.0	<.001	84.8 ± 6.6	71.9 ± 7.3	<.001
SBP (mmHg)	123.6 ± 14.7	111.2 ± 12.0	<.001	121.4 ± 17.1	102.9 ± 12.9	<.001
DBP (mmHg)	80.4 ± 10.5	71.9 ± 9.3	<.001	77.0 ± 11.0	65.7 ± 9.2	<.001
TC (mg/dL)	199.6 ± 36.3	194.8 ± 33.6	<.001	203.9 ± 37.4	187.8 ± 32.9	<.001
TG (mg/dL)	241.2 ± 118.6	131.8 ± 82.8	<.001	190.5 ± 98.3	82.1 ± 44.7	<.001
HDL-C (mg/dL)	43.5 ± 10.1	53.1 ± 11.8	<.001	46.6 ± 9.6	63.8 ± 13.6	<.001
LDL-C (mg/dL)	123.2 ± 32.7	122.1 ± 30.3	.241	130.5 ± 33.8	110.9 ± 29.8	<.001
Glucose (mg/dL)	110.1 ± 30.6	90.2 ± 17.0	<.001	107.4 ± 29.0	85.4 ± 10.9	<.001
HbA1c (%)	6.3 ± 1.2	5.6 ± 0.6	<.001	6.3 ± 1.1	5.4 ± 0.5	<.001
Insulin (μU/mL)	7.9 ± 4.0	4.9 ± 3.0	<.001	7.5 ± 4.2	4.2 ± 2.5	<.001
HOMA-IR	0.4 ± 1.0	0.2 ± 0.5	<.001	0.6 ± 1.1	0.2 ± 0.5	<.001
Uric acid (mg/dL)	6.3 ± 1.5	6.0 ± 1.3	<.001	4.8 ± 1.1	4.2 ± 0.9	<.001
TSH (μIU/mL)	1.89 ± 0.86	1.84 ± 0.85	.037	2.04 ± 0.90	1.99 ± 0.89	.242
FT4 (ng/dL)	1.30 ± 0.15	1.33 ± 0.15	<.001	1.21 ± 0.15	1.23 ± 0.14	.001

Values are presented as means ± standard deviation. BMI = body mass index, DBP = diastolic blood pressure, FT4 = free thyroxine, HbA1c = hemoglobin A1c, HDL-C = high-density lipoprotein-cholesterol, HOMA-IR = homeostatic model assessment for insulin resistance, LDL-C = low-density lipoprotein-cholesterol, MetS = metabolic syndrome, SBP = systolic blood pressure, TC = total cholesterol, TG = triglyceride, TSH = thyroid-stimulating hormone, WC = waist circumference.

### 3.2 Medical characteristics of subjects according to TSH and FT4 quartiles

There were significant differences between groups in body mass index, waist circumference, systolic and diastolic blood pressure, triglyceride, fasting glucose, HbA1c, HOMA-IR, uric acid, TSH, and FT4 according to the TSH and FT4 quartiles in men (Table 2). In women, age, body mass index, waist circumference, total cholesterol, triglyceride, HDL cholesterol, LDL cholesterol, TSH, and FT4 showed significant differences between groups according to the TSH and FT4 quartiles (Table 3). In addition, there was a significant difference in the prevalence of metabolic syndrome according to FT4 quartiles in both men and women (*P* < .001, *P* = .001, respectively), but there was no difference according to TSH quartiles (Tables 2 and 3).

**Table 2:** Anthropometric and biochemical characteristics according to TSH and FT4 quartiles in men.

	TSH (μIU/mL)					FT4 (ng/dL)				
Variables	Q1 (N = 3,050)	Q2 (N = 3,189)	Q3 (N = 3,117)	Q4 (N = 3,122)	*P*	Q1 (N = 2,926)	Q2 (N = 3,195)	Q3 (N = 3,122)	Q4 (N = 3,235)	*P*
Age (years)	46.1 ± 11.0	45.6 ± 10.5	45.5 ± 10.8	45.6 ± 11.0	.130	49.4 ± 11.4	46.6 ± 10.9	44.3 ± 10.2	42.9 ± 9.7	<.001
Weight (kg)	72.1 ± 10.1	72.7 ± 10.2	72.7 ± 10.4	73.0 ± 10.5	.003	72.6 ± 10.3	72.8 ± 10.3	72.8 ± 10.3	72.3 ± 10.3	.113
BMI (kg/m^2^)	24.5 ± 2.9	24.7 ± 2.9	24.7 ± 3.1	24.8 ± 3.0	.038	25.0 ± 3.0	24.8 ± 2.9	24.6 ± 3.0	24.4 ± 3.0	<.001
WC (cm)	83.5 ± 7.6	83.7 ± 7.7	83.8 ± 7.6	84.0 ± 7.6	.047	84.5 ± 7.6	84.0 ± 7.5	83.6 ± 7.6	83.0 ± 7.6	<.001
SBP (mmHg)	111.9 ± 12.5	113.0 ± 12.8	112.6 ± 13.2	113.0 ± 13.5	.001	113.9 ± 13.5	112.4 ± 13.0	112.2 ± 13.1	112.2 ± 12.3	<.001
DBP (mmHg)	72.4 ± 9.7	73.2 ± 9.7	72.9 ± 9.9	73.1 ± 10.1	.006	73.6 ± 10.0	72.8 ± 9.8	72.7 ± 9.9	72.7 ± 9.7	<.001
TC (mg/dL)	193.51 ± 33.7	195.7 ± 33.2	196.2 ± 34.6	195.9 ± 34.3	.007	195.6 ± 35.6	195.3 ± 33.3	195.3 ± 33.6	195.2 ± 33.4	.984
TG (mg/dL)	138.1 ± 90.7	145.3 ± 92.4	145.4 ± 95.5	149.5 ± 98.9	<.001	157.1 ± 111.0	145.5 ± 90.3	143.6 ± 91.1	133.4 ± 83.6	<.001
HDL-C (mg/dL)	52.3 ± 12.2	51.8 ± 11.8	52.1 ± 12.0	51.7 ± 12.1	.116	51.3 ± 12.2	51.7 ± 11.7	52.1 ± 12.1	52.9 ± 12.0	<.001
LDL-C (mg/dL)	121.0 ± 30.8	122.57 ± 29.9	122.8 ± 31.1	122.6 ± 30.5	.065	121.3 ± 31.7	122.6 ± 30.1	122.3 ± 30.2	122.7 ± 30.6	.312
Glucose (mg/dL)	93.0 ± 20.2	93.4 ± 20.8	92.1 ± 20.4	91.7 ± 19.2	.003	93.9 ± 20.4	93.0 ± 19.2	92.0 ± 20.3	91.4 ± 20.7	<.001
HbA1c (%)	5.67 ± 0.74	5.68 ± 0.8	5.63 ± 0.8	5.6 ± 0.7	.003	5.7 ± 0.7	5.7 ± 0.7	5.6 ± 0.8	5.6 ± 0.8	<.001
Insulin (μU/mL)	5.0 ± 3.2	5.5 ± 3.4	5.2 ± 3.2	5.3 ± 3.5	.176	6.1 ± 4.0	5.3 ± 3.2	5.2 ± 3.2	4.6 ± 2.7	<.001
HOMA-IR	0.2 ± 0.5	0.2 ± 0.6	0.2 ± 0.6	0.2 ± 0.7	<.001	0.2 ± 0.7	0.2 ± 0.6	0.2 ± 0.6	0.2 ± 0.5	.003
Uric acid (mg/dL)	6.0 ± 1.3	6.0 ± 1.3	6.0 ± 1.3	6.1 ± 1.3	<.001	6.0 ± 1.3	6.0 ± 1.3	6.1 ± 1.3	6.1 ± 1.3	.013
TSH (μIU/mL)	0.87 ± 0.21	1.43 ± 0.15	2.02 ± 0.19	3.05 ± 0.48	<.001	1.94 ± 0.87	1.86 ± 0.85	1.85 ± 0.85	1.75 ± 0.82	<.001
FT4 (ng/dL)	1.34 ± 0.15	1.34 ± 0.15	1.32 ± 0.15	1.31 ± 0.15	<.001	1.12 ± 0.06	1.27 ± 0.03	1.37 ± 0.03	1.52 ± 0.06	<.001
MetS (n, %)	317 (10.4)	388 (12.2)	372 (11.9)	385 (12.3)	.070	405 (13.8)	389 (12.2)	359 (11.5)	309 (9.6)	<.001

Values are presented as means ± standard deviation. BMI = body mass index, DBP = diastolic blood pressure, FT4 = free thyroxine, HbA1c =  = hemoglobin A1c, HDL-C = high-density lipoprotein-cholesterol, HOMA-IR = homeostatic model assessment for insulin resistance, LDL-C = low-density lipoprotein-cholesterol, MetS = metabolic syndrome, SBP = systolic blood pressure, TC = total cholesterol, TG = triglyceride, TSH = thyroid-stimulating hormone, WC = waist circumference.

**Table 3:** Anthropometric and biochemical characteristics according to TSH and FT4 quartiles in women.

	TSH (μIU/mL)					FT4 (ng/dL)				
Variables	Q1 (N = 1,857)	Q2 (N = 1,914)	Q3 (N = 1,893)	Q4 (N = 1,911)	*P*	Q1 (N = 1,838)	Q2 (N = 1,930)	Q3 (N = 1,822)	Q4 (N = 1,985)	*P*
Age (years)	43.9 ± 11.5	44.1 ± 11.2	44.0 ± 10.9	45.3 ± 11.7	<.001	46.4 ± 11.6	44.7 ± 11.0	43.4 ± 11.0	43.0 ± 11.4	<.001
Weight (kg)	56.3 ± 8.0	56.5 ± 7.2	56.8 ± 8.2	56.8 ± 7.9	.283	57.5 ± 7.9	56.8 ± 8.3	56.4 ± 7.9	55.7 ± 7.8	<.001
BMI (kg/m^2^)	22.4 ± 3.2	22.4 ± 3.2	22.5 ± 3.3	22.6 ± 3.2	.043	23.0 ± 3.2	22.6 ± 3.3	22.3 ± 3.2	22.0 ± 3.1	<.001
WC (cm)	72.3 ± 7.9	72.4 ± 7.8	72.8 ± 7.9	73.3 ± 7.8	<.001	73.7 ± 7.9	73.0 ± 8.0	72.3 ± 7.8	71.8 ± 7.7	<.001
SBP (mmHg)	103.5 ± 13.4	103.8 ± 14.0	104.2 ± 13.9	104.7 ± 14.4	.058	104.5 ± 14.4	103.8 ± 13.6	103.7 ± 13.6	104.3 ± 14.1	.236
DBP (mmHg)	65.9 ± 9.3	66.4 ± 9.6	66.6 ± 9.9	66.7 ± 9.8	.075	66.7 ± 10.0	66.3 ± 9.4	66.2 ± 9.7	66.5 ± 9.7	.261
TC (mg/dL)	185.8 ± 32.9	189.4 ± 34.1	189.4 ± 32.9	190.7 ± 33.5	<.001	190.8 ± 34.1	190.0 ± 33.8	187.1 ± 32.9	187.5 ± 32.7	.001
TG (mg/dL)	85.3 ± 51.3	88.7 ± 54.5	89.6 ± 63.1	92.3 ± 55.7	.002	98.5 ± 60.1	95.0 ± 64.3	83.4 ± 51.8	79.6 ± 45.6	<.001
HDL-C (mg/dL)	62.7 ± 13.8	63.2 ± 14.2	63.0 ± 13.9	62.0 ± 13.9	.046	61.3 ± 14.0	62.2 ± 14.2	63.4 ± 14.0	63.9 ± 13.5	<.001
LDL-C (mg/dL)	109.4 ± 30.0	112.4 ± 31.1	112.3 ± 29.9	114.3 ± 30.6	<.001	114.2 ± 31.2	113.2 ± 30.7	110.5 ± 30.0	110.6 ± 29.7	<.001
Glucose (mg/dL)	88.8 ± 13.4	87.0 ± 14.7	86.9 ± 14.0	86.6 ± 13.6	.898	87.4 ± 14.4	86.6 ± 11.7	86.3 ± 12.6	87.0 ± 16.4	.120
HbA1c (%)	5.5 ± 0.6	5.5 ± 0.6	5.5 ± 0.6	5.5 ± 0.6	.772	5.6 ± 0.6	5.5 ± 0.5	5.5 ± 0.5	5.5 ± 0.6	<.001
Insulin (μU/mL)	4.4 ± 2.8	4.4 ± 2.7	4.6 ± 2.8	4.4 ± 2.7	.743	4.9 ± 3.0	4.6 ± 3.0	4.4 ± 2.8	4.1 ± 2.4	<.001
HOMA-IR	0.2 ± 0.5	0.2 ± 0.6	0.2 ± 0.6	0.2 ± 0.5	.009	0.2 ± 0.6	0.2 ± 0.6	0.2 ± 0.5	0.2 ± 0.5	.306
Uric acid (mg/dL)	4.2 ± 1.0	4.2 ± 0.9	4.2 ± 0.9	4.2 ± 1.0	.718	4.1 ± 1.0	4.2 ± 1.0	4.2 ± 0.9	4.3 ± 1.0	<.001
TSH (μIU/mL)	0.94 ± 0.25	1.59 ± 0.16	2.20 ± 0.20	3.23 ± 0.44	<.001	2.12 ± 0.88	2.11 ± 0.90	1.93 ± 0.86	1.83 ± 0.87	<.001
FT4 (ng/dL)	1.26 ± 0.14	1.23 ± 0.14	1.23 ± 0.14	1.20 ± 0.13	<.001	1.05 ± 0.05	1.17 ± 0.02	1.27 ± 0.02	1.42 ± 0.08	<.001
MetS (n, %)	108 (5.8)	120 (6.3)	125 (6.6)	126 (6.6)	.729	139 (7.6)	141 (7.3)	98 (5.4)	101 (5.1)	.001

Values are presented as means ± standard deviation. BMI = body mass index, DBP = diastolic blood pressure, FT4 = free thyroxine, HbA1c = hemoglobin A1c, HDL-C = high-density lipoprotein-cholesterol, HOMA-IR = homeostatic model assessment for insulin resistance, LDL-C = low-density lipoprotein-cholesterol, MetS = metabolic syndrome, SBP = systolic blood pressure, TC = total cholesterol, TG = triglyceride, TSH = thyroid-stimulating hormone, WC = waist circumference.

### 3.3 Relationship between TSH and FT4 and metabolic syndrome and its components

**Figure 2:**
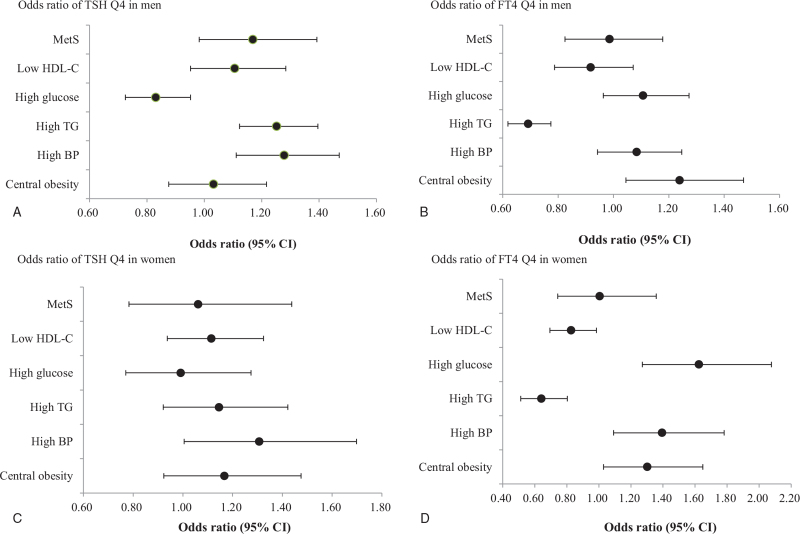
The odds ratio of metabolic syndrome and components of metabolic syndrome according to thyroid function.

TSH was found to have a positive correlation with systolic blood pressure (*P* = .011), diastolic blood pressure (*P* = .026), and triglycerides (*P* = .002) in men, and a negative correlation with fasting glucose (*P* = .003). In women, waist circumference was associated with TSH (*P* < .001). In both men and women, FT4 had a positive association with waist circumference (*P* < .001), systolic blood pressure (*P* = .016, *P* < .001, respectively), diastolic blood pressure (*P* = .004, *P* < .001, respectively), and HDL-cholesterol (*P* = .002, *P* = .016, respectively), and a negative association with triglycerides (*P* < .001) (Table 4). The odds ratio of metabolic syndrome and its components according to TSH and FT4 quartiles in men and women are presented in Tables 5 and 6, respectively. In men, the odds ratio of high blood pressure (OR, 1.278; 95% CI, 1.111–1.470) and hypertriglyceridemia (OR, 1.252; 95% CI, 1.123–1.396) were higher in the fourth quartile than in the first quartile of TSH. For FT4, the odds ratio of abdominal obesity (OR, 1.239; 95% CI, 1.045–1.470) was higher in the fourth quartile than in the first quartile, but the odds ratio of hypertriglyceridemia (OR, 0.692; 95% CI, 0.619–0.774) was low (Table 5, Fig. 2A and B). In women, TSH was associated with a higher odds ratio of high blood pressure (OR, 1.307; 95% CI, 1.005–1.698) in the fourth quartile than in the first quartile. Furthermore, the odds ratio of abdominal obesity (OR, 1.302; 95% CI, 1.029–1.649), high blood pressure (OR, 1.395; 95% CI, 1.092–1.782), and high blood sugar (OR, 1.625; 95% CI, 1.271–2.077) were high in the fourth quartile of FT4, whereas the odds ratio of hypertriglyceridemia (OR: 0.641; 95% CI: 0.512–0.803) was low in the fourth quartile (Table 6, Fig. 2C and D). However, in both men and women, TSH and FT4 levels were not associated with the onset of metabolic syndrome.

**Table 4:** Association between thyroid functions and the components of the metabolic syndrome.

	TSH (μIU/mL)				FT4 (ng/dL)			
	Men		Women		Men		Women	
MetS components	β	*P*	β	*P*	β	*P*	β	*P*
WC (cm)	0.008	.097	**0.020**	**.002**	**0.023**	**<.001**	**0.024**	**<.001**
SBP (mm Hg)	**0.022**	**.011**	0.016	.120	**0.021**	**.016**	**0.070**	**<.001**
DBP (mm Hg)	**0.019**	**.026**	0.012	.243	**0.026**	**.004**	**0.060**	**<.001**
TG (mg/dL)	**0.027**	**.002**	0.014	.187	**−0.080**	**<.001**	**−0.081**	**<.001**
HDL-C (mg/dL)	−0.006	.474	−0.008	.496	**0.027**	**.002**	**0.027**	**.016**
Glucose (mg/dL)	**−0.026**	**.003**	−0.015	.160	0.010	.264	**0.039**	**<.001**

Adjusted for age and body mass index. DBP = diastolic blood pressure, FT4 = free thyroxine, HDL-C = high density lipoprotein cholesterol, MetS = metabolic syndrome, SBP = systolic blood pressure, TG = triglyceride, TSH = thyroid stimulating hormone. *β*, Standardized beta coefficient.

**Table 5:** Risk of metabolic syndrome and components of metabolic syndrome according to thyroid function quartiles in men.

TSH (μIU/mL)	Q1	Q2 OR (95% CI)	Q3 OR (95% CI)	Q4 OR (95% CI)
Central obesity	1.00	1.004 (0.852–1.183)	0.990 (0.838–1.170)	1.032 (0.876–1.217)
High BP	1.00	**1.264 (1.099–1.454)**	**1.191 (1.033–1.372)**	**1.278 (1.111–1.470)**
High TG	1.00	**1.165 (1.045–1.299)**	**1.140 (1.022–1.272)**	**1.252 (1.123–1.396)**
High glucose	1.00	0.989 (0.866–1.130)	0.876 (0.765–1.004)	0.831 (0.725–0.952)
Low HDL-C	1.00	1.039 (0.894–1.208)	0.958 (0.821–1.117)	1.106 (0.952–1.284)
MetS	1.00	1.184 (0.995–1.408)	1.119 (0.938–1.335)	1.169 (0.982–1.392)
FT4 (ng/dL)	Q1	Q2 OR (95% CI)	Q3 OR (95% CI)	Q4 OR (95% CI)
Central obesity	1.00	**1.245 (1.059–1.465)**	**1.227 (1.039–1.451)**	**1.239 (1.045–1.470)**
High BP	1.00	0.952 (0.831–1.090)	1.051 (0.915–1.207)	1.084 (0.942–1.247)
High TG	1.00	**0.860 (0.772–0.958)**	**0.862 (0.772–0.961)**	**0.692 (0.619–0.774)**
High glucose	1.00	1.090 (0.955–1.243)	1.067 (0.930–1.224)	1.107 (0.963–1.273)
Low HDL-C	1.00	0.958 (0.826–1.111)	0.924 (0.794–1.078)	0.918 (0.787–1.071)
MetS	1.00	1.050 (0.889–1.241)	1.097 (0.925–1.302)	0.986 (0.825–1.178)

Adjusted odds ratios for age and body mass index. BP = blood pressure, CI = confidence interval, FT4 = free thyroxine, HDL-C = high density lipoprotein cholesterol, MetS = metabolic syndrome, OR = odds ratio, TG = triglyceride, TSH = thyroid stimulating hormone.

**Table 6:** Risk of metabolic syndrome and components of metabolic syndrome according to thyroid function quartiles in women.

TSH (μIU/mL)	Q1	Q2 OR (95% CI)	Q3 OR (95% CI)	Q4 OR (95% CI)
Central obesity	1.00 (Reference)	1.071 (0.839–1.366)	1.199 (0.943–1.525)	1.167 (0.923–1.475)
High BP	1.00 (Reference)	1.146 (0.871–1.506)	**1.484 (1.139–1.933)**	**1.307 (1.005–1.698)**
High TG	1.00 (Reference)	1.004 (0.802–1.258)	1.051 (0.840–1.314)	1.145 (0.921–1.422)
High glucose	1.00 (Reference)	1.145 (0.890–1.472)	1.010 (0.781–1.305)	0.991 (0.770–1.274)
Low HDL-C	1.00 (Reference)	1.019 (0.854–1.215)	1.035 (0.868–1.234)	1.114 (0.937–1.324)
MetS	1.00 (Reference)	1.152 (0.846–1.568)	1.198 (0.882–1.626)	1.061 (0.783–1.438)
FT4 (ng/dL)	Q1	Q2 OR (95% CI)	Q3 OR (95% CI)	Q4 OR (95% CI)
Central obesity	1.00 (Reference)	**1.373 (1.091–1.728)**	1.255 (0.991–1.590)	**1.302 (1.029–1.649)**
High BP	1.00 (Reference)	0.855 (0.661–1.107)	1.001 (0.770–1.299)	**1.395 (1.092–1.782)**
High TG	1.00 (Reference)	1.088 (0.892–1.327)	**0.656 (0.524–0.823)**	**0.641 (0.512–0.803)**
High glucose	1.00 (Reference)	1.129 (0.878–1.453)	1.211 (0.934–1.568)	**1.625 (1.271–2.077)**
Low HDL-C	1.00 (Reference)	1.001 (0.847–1.182)	0.903 (0.758–1.074)	0.827 (0.694–0.985)
MetS	1.00 (Reference)	1.241 (0.938–1.641)	0.999 (0.738–1.352)	1.005 (0.743–1.358)

Adjusted odds ratios for age and body mass index BP = blood pressure, CI = confidence interval, FT4 = free thyroxine, HDL-C = high density lipoprotein cholesterol, MetS = metabolic syndrome, OR = odds ratio, TG = triglyceride, TSH = thyroid stimulating hormone.

## 4 Discussion

The present study, which was conducted among euthyroid participants in South Korea, demonstrated that FT4 was inversely correlated with triglycerides in both men and women, and that a decrease in FT4 levels was associated with an increase in the prevalence of hypertriglyceridemia. In addition, in both men and women, TSH was associated with an increased incidence of elevated blood pressure, and FT4 was associated with an increased prevalence of abdominal obesity. However, after correcting for age and body mass index in both men and women, the relationship between metabolic syndrome and thyroid hormones could not be confirmed.

Thyroid hormones play an important physiological role in maintaining blood sugar, controlling appetite, regulating sympathetic nerve activity, energy homeostasis, and metabolism.^[22]^ Hypothyroidism is associated with low energy expenditure and fluid retention, while hyperthyroidism is known to cause weight loss due to increased energy demand.^[23,24]^ Moreover, thyroid dysfunction is accompanied by glucose transport disorder and insulin resistance. Consequently, it induces or worsens metabolic syndrome.^[8,25,26]^ Therefore, it has been reported that thyroid dysfunction and metabolic syndrome are associated with cardiovascular morbidity and mortality.^[27,28]^ However, the relationship between thyroid hormone function level within the normal range and metabolic disease has not been fully elucidated.^[29]^

Waring et al^[30]^ reported that, in the elderly euthyroid population, TSH was related to triglycerides and HDL-cholesterol level and that higher TSH levels were associated with a higher prevalence of metabolic syndrome. By contrast, Kim et al^[31]^ found a relationship between TSH and body mass index, HDL-cholesterol, fasting glucose, and HbA1c in middle-aged euthyroid individuals but reported no association with metabolic syndrome. Furthermore, Roos et al^[32]^ suggested that FT4 was related to blood pressure and that FT4 was a risk factor for cardiovascular disease.

When the results of this study were stratified by sex, the odds ratio high blood pressure was found to be higher in the fourth quartile than in the first quartile of TSH, after adjusting for age and body mass index, in both men and women. In addition, the odds ratio of abdominal obesity was significantly higher, whereas the odds ratio of hypertriglyceridemia was significantly lower in the fourth quartile than in the first quartile of FT4 in both men and women. Therefore, this suggests study that even if the thyroid function level is within the normal range, high TSH and FT4 levels may increase the prevalence of high blood pressure and abdominal obesity, respectively. On the contrary, low FT4 levels may increase the prevalence of hypertriglyceridemia. Jang et al^[33]^ reported a negative association between FT4 and triglycerides in 1423 participants aged 10 years or older using the 6th National Health and Nutrition Survey in South Korea from 2013 to 2015, which is consistent with our results.

According to a study conducted among Koreans, in euthyroid menopausal women, TSH was associated with triglycerides, and the OR for metabolic syndrome was 1.9 in the fourth quartile, compared with that in the first quartile of TSH.^[34]^ However, low FT4 levels within the reference range have also been associated with metabolic syndrome, but this association was not significant after correcting for age.^[13]^ Therefore, inconsistent results have been reported on the association between thyroid function and metabolic syndrome in euthyroid subjects.^[31,32,35]^ Despite the association of thyroid hormone function with metabolic syndrome components, we could not confirm its association with metabolic syndrome. This discrepancy is thought to be due to differences in study groups, such as age and sex of the participants, iodine intake, other genetic and environmental factors, and factors included in the correction during statistical analysis.

The mechanisms underlying the association between thyroid function and the components of metabolic syndrome are not fully understood. Stimulation of the sympathetic nerve by thyroid hormones affects not only glucose and lipid metabolism but also cardiovascular regulation.^[8,22,36]^ Hyperglycemia is caused by a decrease in glucose uptake to the muscles in hypothyroidism, and it is caused by an increase in hepatic glucose production in the liver in hyperthyroidism.^[37]^ In addition, thyroid hormone stimulates lipid synthesis and decomposition, the relationship with dyslipidemia can be inferred.^[38]^ Specifically, the negative association between triglycerides and thyroid hormones is presumed to be due to an increase in the removal rate of triglycerides from plasma due to an increase in the activity level of hepatic triglyceride lipase.^[39,40]^

In this study, there was a gender difference in the association between serum TSH and FT4 and metabolic syndrome components in euthyroid subjects, because sex hormones affect thyroid function and show gender differences in metabolic syndrome expression patterns.^[41,42]^

The strength of this study is that the analysis was performed based on a large Korean population, and the relationship between TSH and FT4 levels and metabolic syndrome was evaluated according to sex. However, this study has several limitations. First, as a cross-sectional study, it was not possible to infer a causal relationship between TSH and FT4 and metabolic syndrome in euthyroid subjects. Large longitudinal cohort studies and randomized clinical trials are required to confirm this association. Second, this study did not consider drinking, smoking, physical activity, and the presence or absence of menopause in women that affect thyroid function and metabolic syndrome. Third, research results are reported that the prevalence of diabetes can cause changes in lipid profiles, and there is a limitation in that subjects with metabolic syndrome were divided into a previously diagnosed group and a recently diagnosed group, and the relationship could not be confirmed.^[43,44]^

## 5 Conclusions

There was a significant relationship between serum TSH and FT4 levels and different metabolic syndrome components according to the sex of participants with normal thyroid function. However, despite this finding, we confirmed that there was no association between TSH and FT4 levels and metabolic syndrome.

## Author contributions

**Conceptualization:** Eun Jae Kim.

**Data curation:** Eun Jae Kim.

**Formal analysis:** Eun Jae Kim.

**Investigation:** Kyung A. Shin, Eun Jae Kim.

**Methodology:** Kyung A. Shin, Eun Jae Kim.

**Supervision:** Kyung A. Shin.

**Visualization:** Eun Jae Kim.

**Writing – original draft:** Kyung A. Shin.

**Writing – review & editing:** Kyung A. Shin.
